# Annual Report of the Royal Veterinary Institute, Berlin, Prussia, for the Year 1882-’3

**Published:** 1884-01

**Authors:** 


### Annual Report of the Royal Veterinary Institute, Berlin, Prussia, for the year 1882-'83.

179 Students attended the summer course 1882.
247 " " " winter " 1882-'83.

Of the fifty students that presented themselves for the final examination, Easter, 1882—only two passed as very good fifteen as "good," and twenty-two as "satisfactory."

Fifty-four horses and numerous parts of horses and animals were purchased for anatomical purposes during the winter session, 1882-'83.

The horses cost forty marks ($10.00) and are difficult to to procure at that price.

*Horse Hospital Report.*

| | | |
|---|---|---|
| Treated for various diseases, in hospital | 1,918 | horses. |
| Examinations for soundness | 703 | " |

*Poli-Clinic.*

| | | |
|---|---|---|
| Treated for disease | 4,660 | " |
| Examined (no certificate given) | 163 | " |
| Total | 7,444 | |

*Hospital for Smaller Domestic Animals.*

| | | |
|---|---|---|
| Treated in hospital | 990 | animals. |
| Treated in free-clinic | 3,913 | |
| Total | 4,903 | |
| Total number of animals treated at the hospital during the year 1882-'83—(from Easter to Easter.) | 12,347 | |
| Autopsies on horses at the Pathological Institute | 145 | |

Numerous others were made on the smaller animals.

The students visited outside the school, on account of infectious or contagious disease, 15 herds of cattle, 5 flocks of cattle, 6 droves of cattle; on account of sproadic disease, 430 head of cattle, besides sheep, goats, and other animals.

